# Microbial network-driven remediation of saline-alkali soils by salt-tolerant plants

**DOI:** 10.3389/fmicb.2025.1565399

**Published:** 2025-04-09

**Authors:** Yushuang Cui, Zhifang Ning, Menglu Li, Xue Qin, Xin Yue, Xiaobo Chen, Changxiong Zhu, Hongyong Sun, Yali Huang

**Affiliations:** ^1^College of Environmental Science and Engineering, Hebei University of Science and Technology, Shijiazhuang, China; ^2^College of Food Science and Biology, Hebei University of Science and Technology, Shijiazhuang, China; ^3^Center for Agricultural Resources Research, Institute of Genetics and Developmental Biology, Chinese Academy of Sciences, Shijiazhuang, China

**Keywords:** salt-tolerant plants, saline-alkali soil remediation, network analysis, microbial community structure, keystone taxa

## Abstract

Salt-tolerant plants (STPs) play an important role in saline-alkali soil remediation, but their interaction with soil microorganisms remain incompletely elucidated. This study explored the effects on microbial community structure, function, and soil quality in saline-alkali land of four treatments: no plant (CK), *Triticum aestivum* L. (TA), *Tamarix chinensis* Lour. (TC), and *Hibiscus moscheutos* Linn. (HM). The results indicated that the planting of TC, TA, and HM effectively reduced soil electrical conductivity (EC) by 82.9, 88.3, and 86.2%, respectively. TC and TA significantly decreased the pH from 8.79 to 8.35 and 8.06, respectively, (*p* < 0.05). Moreover, the nutrient content and enzymatic activities were enhanced. Notably, TA exhibited the most significant soil nutrient improvement. STPs also substantially altered the microbial community structure and function, with TC increasing bacterial richness (ACE and Chao1 indices) compared to other treatments (*p* < 0.05). Moreover, TA significantly promoted the relative abundance of *unclassified_Gemmatimonadaceae*, *unclassified_Vicinamibacterales*, and *Mortierella* (*p* < 0.05). A major innovation of this study is using network analysis to explore microbial interactions, revealing how STPs enhance microbial network complexity. This approach identified *Sphingomonas* as a key taxon in TA soils, shedding light on the microbial dynamics of soil remediation. Additionally, partial least squares path model (PLS-PM) showed that soil quality improvements were primarily driven by shifts in bacterial composition, offering a novel mechanistic framework for understanding microbial contributions to soil restoration. This research advances the understanding of microbial-plant interactions and underscores the innovative application of network analysis in phytoremediation, offering valuable insights for future soil restoration strategies.

## 1 Introduction

By 2050, it is predicted that nearly 50% of global arable land will be affected by salinization which characterized by high electrical conductivity, reduced water potential, and excess ionic salts, posing a significant threat to agricultural productivity (Hafez et al., 2022a; Jia et al., 2023). To mitigate these challenges, various rehabilitation methods have been employed (Hafez et al., 2022b; Gao et al., 2024; Youssef et al., 2024) among which phytoremediation using salt-tolerant plants (STPs) has emerged as a sustainable and effective approach (Li et al., 2023; Waheed et al., 2024).

Recent studies highlight that the resilience of plants to salt stress is strongly influenced by the interactions between plants and their associated microbial communities (Hafez et al., 2020; Zheng et al., 2021) Bacteria colonizing the roots of STPs can help plants acclimate to salt stress by improving nutrient absorption or secreting growth-promoting compounds (Ilangumaran and Smith, 2017; Zhou et al., 2024). For example, the introduction of *Bacillus* sp. *MT7* to the tomato rhizosphere has been shown to boost the salt tolerance of tomatoes (Pathania et al., 2020). These findings collectively emphasize the critical role of microorganisms in improving saline-alkali soils through the cultivation of STPs.

Soil microorganisms form complex communities, dominated by a few abundant taxa and supplemented by a vast array of rare taxa. How STPs affect microbial community structure remains a subject of ongoing interest. STPs can enrich beneficial bacteria in the soil by releasing organic acids and enzymes that promote plant growth under saline stress (Zahra et al., 2024). For instance, Shao et al. (2019) showed that Jerusalem artichoke enhances microbial diversity in saline-alkali soils through root exudates in Jiangsu Province. Additionally, different STP species release varying organic compounds, which attract beneficial rhizosphere bacteria under saline stress, thereby aiding the host plant in mitigating salt-induced damage (Xiong et al., 2020). Despite these advances, the microbiota of diverse STPs in saline soils remains underexplored, particularly with regard to keystone taxa. These taxa, which serve as central components of the core microbiome, play a critical role in facilitating plant–soil interactions and enhancing soil improvement (Trivedi et al., 2020; Liu Z. et al., 2023). Therefore, understanding how different STPs improve saline-alkali soils and identifying keystone taxa associated with each STP could pave the way for targeted microbial selection and the construction of keystone taxa networks.

In this study, we utilized three different STPs—*Triticum aestivum* L., *Tamarix chinensis* Lour., and *Hibiscus moscheutos* Linn.—to restore saline-alkali soil. Our objectives were: (i) to evaluate the effects of different STPs on soil nutrient content and enzyme activity; (ii) to examine changes in microbial community structure and network complexity in saline-alkali soils treated with various STPs, identifying the keystone taxa associated with saline-alkali tolerance; and (iii) to analyze the interactions between microbial composition and soil properties. The innovation of this article lies in the integrated use of microbial network analysis, functional prediction analysis to reveal the multi-dimensional impacts of salt-tolerant plants on the structure, function of microbial communities, and soil quality in saline-alkali lands. The paper provides new insights for the remediation of saline-alkali lands.

## 2 Materials and methods

### 2.1 Experimental design and soil sampling

The on-site experimental study was conducted in Haixing County, Hebei Province, China, within a research area of the Chinese Academy of Sciences dedicated to the efficient use of coastal saline soils (117°33′49′′E, 38°10′02′′N). Haixing County, located in Cangzhou City, Hebei Province, is characterized by extensive saline-alkali soils, largely due to high groundwater salinity and limited freshwater resources, leading to severe land salinization. A uniform coastal zone was selected for the experiment to ensure consistent soil characteristics across all plots. The saline-alkali soil in Haixing County was classified as Solonchaks according to the World Reference Base for Soil Resources (WRB), characterized by high salinity accumulation (EC > 4 dS/m) and alkaline pH. During the soil sampling period, Haixing County experiences an average temperature of around 15°C, with precipitation averaging 30–50 millimeters. The climate is dry, characterized by strong southeast winds and humidity levels ranging from 50 to 60%.

The field planting experiment began in 2019 and included four treatments: (i) barren land with no plant (CK); (ii) land cultivated with *Hibiscus moscheutos* Linn. (HM); (iii) land cultivated with *Tamarix chinensis* Lour. (TC); and (iv) land cultivated with *Triticum aestivum* L. (TA). Each treatment has three replicates. These species represent herbaceous, shrub, and ornamental plant types, covering distinct ecological niches, thereby enabling a comprehensive evaluation of diverse STPs remediation potential. Furthermore, all selected species are well-adapted to the climatic and soil conditions of the study area.

Soil samples for each treatment were taken during April 2023. Within each plot area, five soil cores were selected randomly and combined to create a single composite sample. The composite samples were mixed evenly and filtered via a 2-mm sieve for the removal of plant remnants and roots. The samples were then separated into three portions: one portion was air-dried and finely ground to assess chemical properties and enzyme activity; a second portion was stored at 4°C to determine the abundance of culturable soil microorganisms; and a third portion was stored at −80°C for high-throughput sequencing.

### 2.2 Soil physical and chemical property analysis

The pH of the soil was determined in a soil-to-water suspension at a 1:2.5 proportion by means of a pH meter (PB-10, Beijing, China). Electrical conductivity (EC) was determined with a conductivity meter (DDS-307A, Shanghai Leici, China) at a soil-to-water ratio of 1:5. Soil organic matter (SOM) was assessed using the potassium dichromate oxidation method. Total nitrogen (TN), total potassium (TK), total phosphorus (TP), available nitrogen (AN), available phosphorus (AP), and available potassium (AK) were measured according to previously described methods (Yan et al., 2021).

### 2.3 Enzyme activity assays in soil

Catalase (Cat) activity was assessed with KMnO titration. Urease (Ure) activity was measured via the phenol-sodium hypochlorite colorimetric approach. Invertase (Inv) activity was measured by colorimetry using 3,5-dinitrosalicylic acid, and alkaline phosphatase (ALP) activity was assessed using the phosphomolybdic benzidine colorimetric method (Zhao et al., 2024).

### 2.4 DNA extraction, amplification, and sequencing

Genomic DNA of soil samples was isolated with the TGuide S96 magnetic bead approach (model DP812) soil genomic DNA extraction kit from Tiangen Biochemical Technology Co., Ltd. The amplification of the bacterial 16S rRNA gene (V3–V4 region) was performed with the primers 338F (5′-ACTCCTACGGGAGGCAGCA-3′) and 806R (5′-GGACTACHVGGGTWTCTAAT-3′). For fungi, the ITS1 region of the internal transcribed spacer (ITS) was amplified with the primers ITS1F (5′-CTTGGTCATTTAGAGGAAGTAA-3′) and ITS2 (5′-GCTGCGTTCTTCATCGATGC-3′). The PCR products were subjected to purification using a DNA gel extraction kit (manufactured by Axygen in Shanghai, China), and then examined through 1.8% agarose gel electrophoresis. High-throughput sequencing was subsequently carried out on an Illumina HiSeq 6,000 platform at Biomarker Technologies Corp. Raw data were processed into sequence reads utilizing Base Calling software, and the resulting sequences and quality metrics were archived in FASTQ format.

### 2.5 Construction and analysis of microbial co-occurrence networks

The co-occurrence network was built by following the protocol accessible at http://ieg4.rccc.ou.edu/MENA/. ASVs present in less than 0.01% of sample occurrences were excluded. According to within-module connectivity (Zi) and among-module connectivity (Pi), node roles in microbial networks were classified into four categories: module hubs (nodes with high connectivity within modules, Zi > 2.5 and Pi < 0.62), and connectors (nodes that link modules, Zi < 2.5 and Pi > 0.62) network hubs (highly connected nodes within the entire network, Zi > 2.5 and Pi > 0.62), and peripherals (nodes with few connections outside their modules, Zi < 2.5 and Pi < 0.62; Hu et al., 2024). Module hubs, connectors, and network hubs were identified as the keystone taxa. Network visualizations were generated using Gephi 0.10.1.

### 2.6 Statistical and data analysis

Quantitative Insights into Microbial Ecology (QIIME) software was used to filter, splice, and remove chimeras from the raw sequence data obtained by high-throughput sequencing. Operational taxonomic units (ASVs) were generated, and representative sequences for each ASV were selected, aligned, and annotated against the Greengenes database. The alpha diversity measures of soil fungal and bacterial populations were calculated by means of the “vegan” package within R 4.0.3 (Liu Z. et al., 2023). Microbial composition was examined via Principal Coordinate Analysis (PCoA) founded on binary Jaccard distances (Zheng et al., 2023). Functional predictions of bacterial communities was performed by the PICRUSt2 method. Functional profiling of fungal communities was performed utilizing the FUNGuild approach. The associations between bacterial and fungal diversity and soil physicochemical factors were evaluated via Mantel tests with the ecodist package in R (Wen et al., 2024). A partial least squares path model (PLS-PM) was employed to ascertain the direct or indirect impacts of diverse STPs on soil quality and microbial community. The STPs (CK and all treatments were assigned 0 and 1, respectively) were treated as the exogenous variable (Liu et al., 2022). The soil enzyme activity, culturable microorganisms, and physicochemical indicators data were analyzed using Origin and Excel software. Single factor analysis of variance (ANOVA) was performed on the experimental data with SPSS statistical analysis software. Significance test was conducted by Duncan method (*p* < 0.05).

## 3 Results and analysis

### 3.1 Impact of phytoremediation on soil chemical properties and enzyme activity

Table 1 presents the soil’s chemical and physical properties. TC and TA significantly decreased the pH from 8.79 to 8.35 and 8.06, respectively, (*p* < 0.05). TC, TA, and HM effectively reduced the EC by 83, 88, and 86%, respectively. These results indicated that all three plants had the ability to reduce soil pH and salinity (*p* < 0.05). In addition, TA showed the best effect on soil pH and salinity decrease among the three plants.

**Table 1:** Impact of different salt-tolerant plants (STPs) on soil physicochemical properties.

	CK	HM	TC	TA
pH	8.79 ± 0.04a	8.84 ± 0.01a	8.35 ± 0.02b	8.06 ± 0.04c
EC (μS/cm)	4633.33 ± 326.24a	641.33 ± 15.0b	791.00 ± 13.0b	540.33 ± 16.2b
TN (g/kg)	0.44 ± 0.01d	0.69 ± 0.01c	0.91 ± 0.01b	1.07 ± 0.02a
TP (g/kg)	0.58 ± 0.01d	0.60 ± 0.01c	0.93 ± 0.01b	1.40 ± 0.01a
TK (g/kg)	18.35 ± 0.27c	19.40 ± 0.11b	20.00 ± 0.12a	20.27 ± 0.22a
AN (mg/kg)	31.09 ± 1.4d	46.44 ± 2.28c	70.76 ± 1.9b	95.43 ± 1.73a
AP (mg/kg)	3.19 ± 0.25c	1.67 ± 0.15c	34.65 ± 1.5b	74.63 ± 2.1a
AK (mg/kg)	152.50 ± 7.0c	194.83 ± 5.5b	233.33 ± 7.6a	159.50 ± 2.6c
SOM (g/kg)	8.26 ± 0.32d	11.69 ± 0.4c	16.83 ± 0.1b	17.73 ± 0.2a

Diferent lowercase letters indicate signifcant diferences, ANOVA, Duncan test, *p* < 0.05. The letter sequence (a > ab > b > bc > c) corresponds to descending order of mean values.

**Figure 1:**
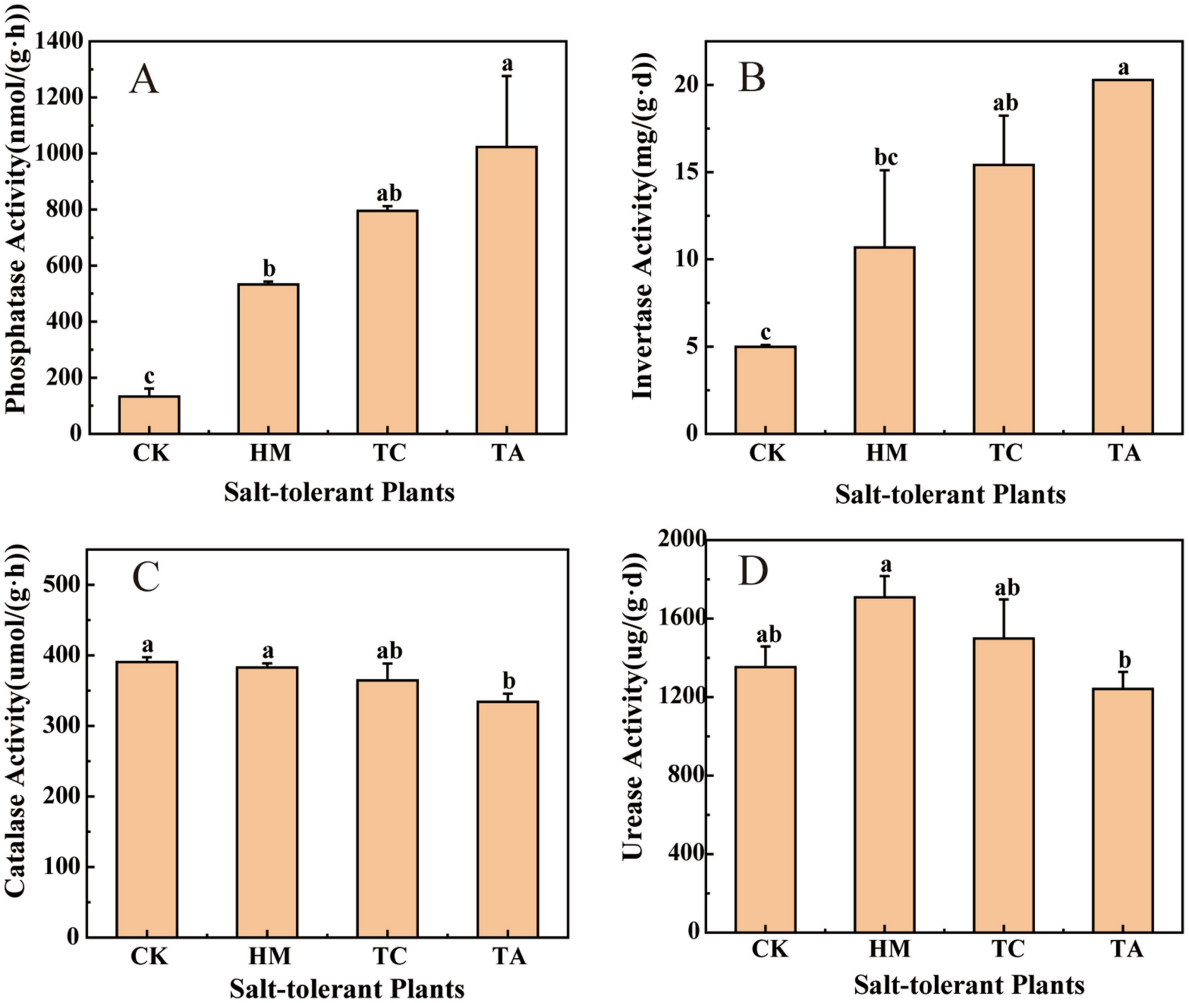
Effects of different STPs on soil enzyme activity, **(A)** alkaline phosphatase, **(B)** invertase, **(C)** catalase, **(D)** urease. Diferent lowercase letters indicate signifcant diferences, ANOVA, Duncan test, *p* < 0.05. The letter sequence (a > ab > b > bc > c) corresponds to descending order of mean values.

The planting of three kinds of plants significantly increased soil fertility related indexes. Compared with CK, HM, TC and TA significantly increased TN by about 57, 107 and 143%, TP by about 3, 55 and 141%, TK by about 6, 9 and 10%, and SOM by about 42, 104 and 115% (*p* < 0.05). TC and TA significantly increased AP content by about 10 and 23 times, respectively. HM and TC treatments significantly increased AK content by about 28 and 53%, respectively. These results showed that phytoremediation significantly improved soil nutrient levels and organic matter content, and TA treatment had the most significant effect on soil fertility. The results of soil enzyme activity are shown in Figure 1. The three STPs significantly increased soil alkaline phosphatase and invertase activities. These results indicate that salt-tolerant plants have the ability to improve soil fertility while reducing soil pH and salinity.

### 3.2 Phytoremediation-induced changes in microbial community structure

Illumina MiSeq sequencing generated 960,232 16S rDNA sequences related to bacteria and 959,820 ITS sequences for fungi within the four treatment groups. Subsequently, we grouped these sequences into an ASV table, ensuring 100% sequence similarity. As the result, it was observed that a total of 15,777 ASVs were detected in the four samples. Among these, there were 119, 129, and 138 ASVs shared by CK with HM, TA, and TC, respectively. There were 3,569, 2,485, 2,477, and 2,580 unique ASVs in CK, HM, TA, and TC, respectively. A similar trend was found for fungi as well. There were 66, 41, and 47 ASVs shared between CK and HM, TA, and TC, respectively. However, there were 931, 963, 944, and 910 unique ASVs in CK, HM, TA, and TC, respectively, (Supplementary Figure S1).

**Figure 2:**
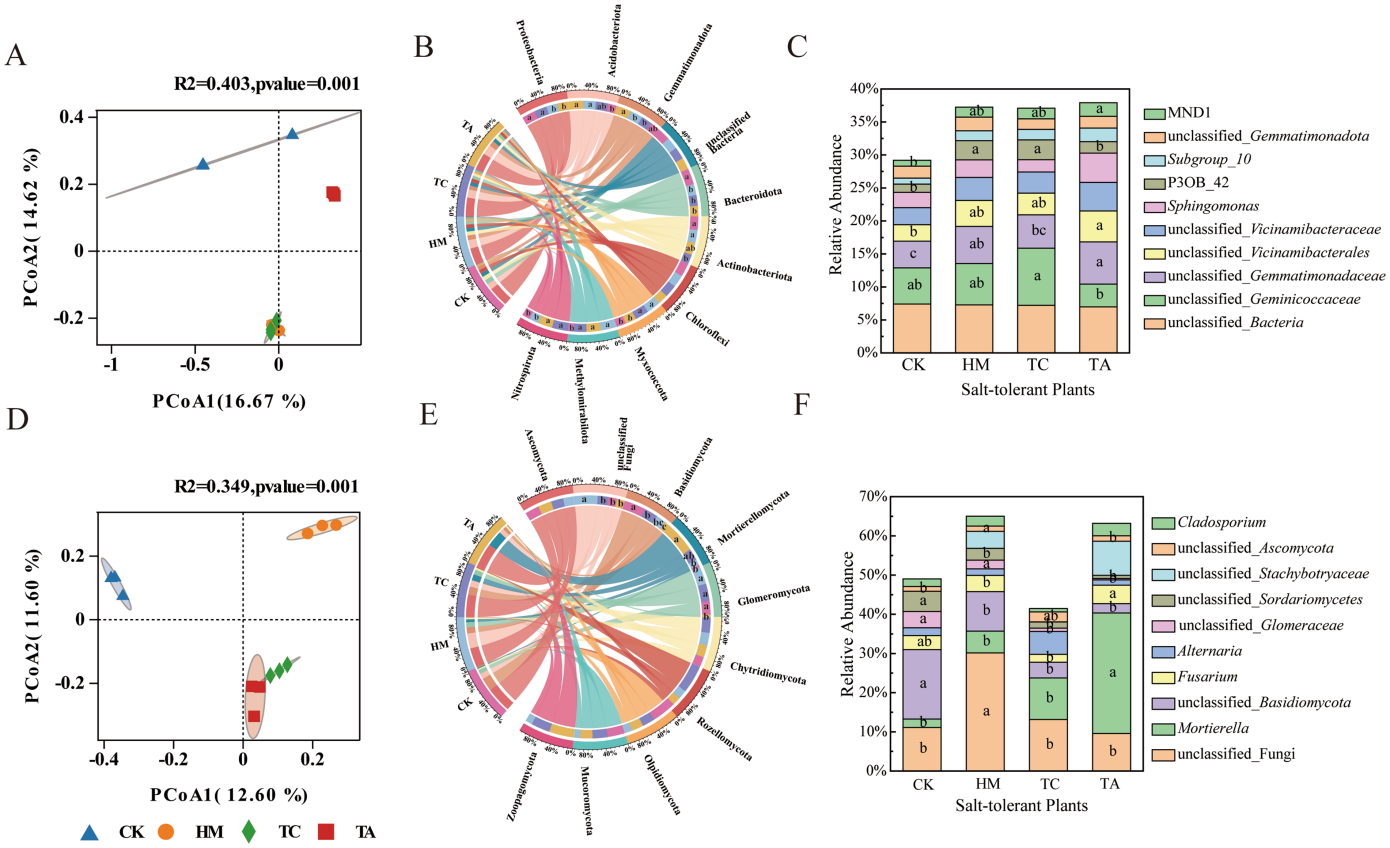
Changes in bacterial and fungal communities: **(A)** bacterial *β*-diversity, **(B)** bacterial community structure at the phyla level, **(C)** bacterial community structure at the genus level; **(D)** fungal *β*-diversity, **(E)** fungal community structure at the phyla level, **(F)** fungal community structure at the genus level.

The impact of different STPs on fungal and bacterial *α*-diversity in soil was assessed (Supplementary Figure S2). Among the three STPs, only TC significantly increased the bacterial species richness indices (ACE and Chao1) (*p* < 0.05).These results indicated that the introduction of STPs had minimal effect on microbial species richness and diversity in saline-alkali soil. PCoA statistics indicated the bacterial and fungi communities associated with HM, TC, and TA were clearly separated from CK, indicating that the introduction of STPs significantly altered soil bacterial and fungi community composition (*p* = 0.001; Figure 2). Three STPs were located close together, suggesting a similarity in the microorganism communities associated with these plants.

The effects of the three STPs on bacterial phyla were analyzed (Figure 2B). Considerable distinctions existed in the manner by which the three STPs affected the relative abundance of the dominant bacterial phyla within the saline-alkali soil. There were notable differences in how the three STPs influenced the relative abundance of the dominant bacterial phyla within saline-alkali soil. Compared to CK, both HM and TA markedly decreased the abundance of Proteobacteria whereas increasing the abundance of Acidobacteriota (*p* < 0.05). At the genus level, the relative abundance of unclassified_*Gemmatimonadaceae* and unclassified_*Vicinamibacterales* in TA soil, increased significantly (*p* < 0.05) compared to CK. Additionally, the relative abundance of P3OB_42 in TC soil was markedly greater than that in CK (*p* < 0.05; Figure 2C).

Ascomycota (44.52%), unclassified_Fungi (16.00%), Basidiomycota (14.91%) and Mortierellomycota(12.78%), were the dominant fungal phyla. Compared to CK, the Basidiomycota were significantly lower in three STPs and the unclassified_Fungi in HM and Mortierellomycota in TA was notably higher (*p* < 0.05; Figure 2E). For the fungial at genus level, HM significantly enriched unclassified_fungi, while TA significantly enriched *Mortierella* (Figure 2F). All STPs significantly reduced the abundance of unclassified_*Basidiomycota*.

### 3.3 Modifications in soil microbial network complexity following phytoremediation

**Figure 3:**
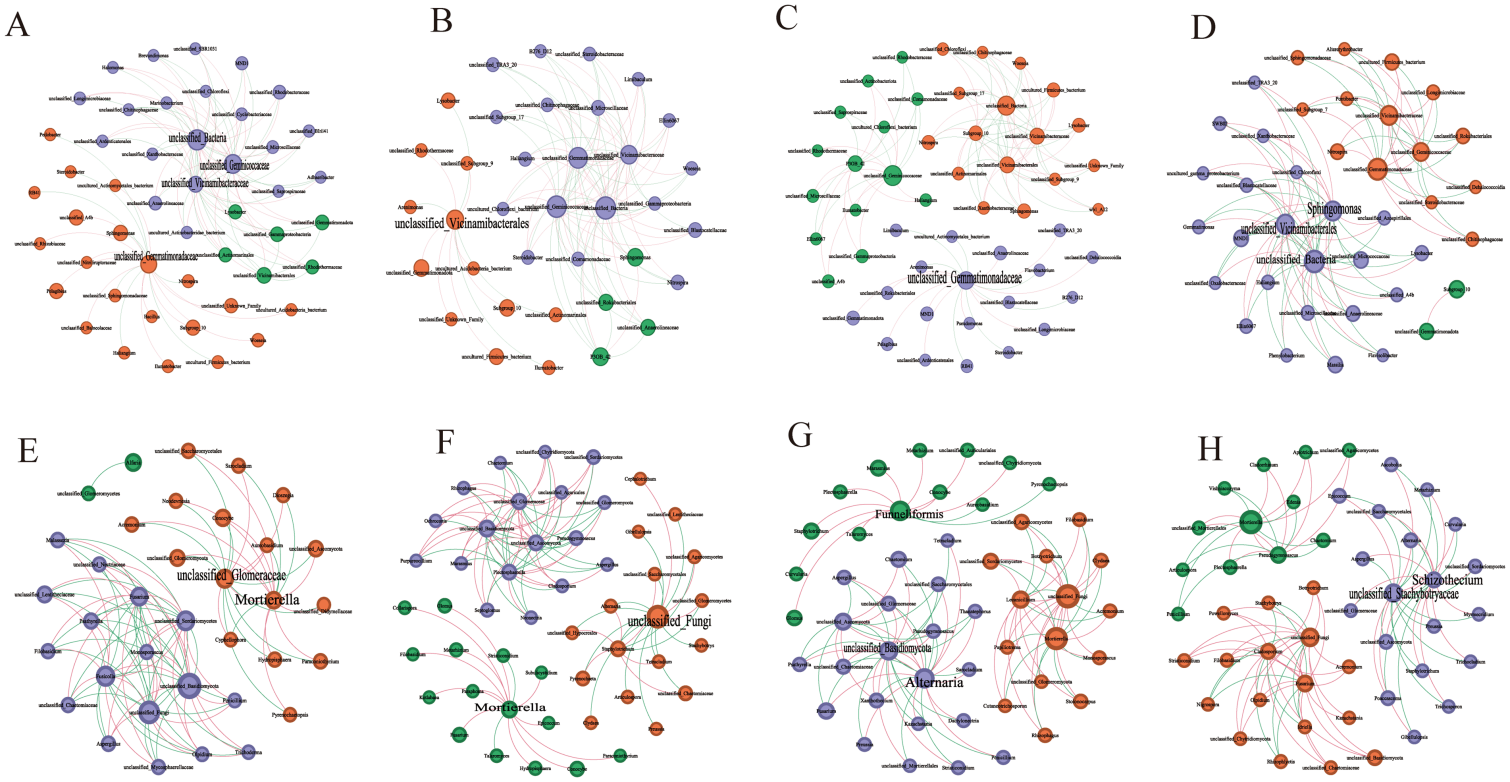
Microbial network interactions in different STP soil samples: **(A)** bacteria in CK, **(B)** bacteria in HM, **(C)** bacteria in TC, **(D)** bacteria in TA; **(E)** fungi in CK, **(F)** fungi in HM, **(G)** fungi in TC, **(H)** fungi in TA.

Table 2 and Figure 3 illustrate the differences in the symbiotic patterns of fungi and bacteria across various plant soils. Compared to CK, all three plant soils reduced the average shortest path length of bacteria network. While, both HM and TA notably decreased the number of bacterial nodes and increased the network density. Additionally, the bacterial modularity in TC and TA was higher than that in CK, while it was lower in HM. In contrast, the three STPs increased the number of fungal nodes, the average shortest path length, and modularity, while decreasing network density and the average clustering coefficient. These results suggest that STPs enhance the size, complexity, and tightness of fungal communities, thereby improving their resistance to environmental fluctuations. In the fungal network of plant soil, positive interactions were strengthened and negative correlations were attenuated, indicating that phytoremediation promotes cooperation among the fungal community. In summary, the effects of the three STPs on bacterial networks were different, while the effects on fungal networks were similar.

**Table 2:** Topological properties of co-occurring networks in different STP soil samples.

Network metrics	Bacteria				Fungi			
	CK	HM	TC	TA	CK	HM	TC	TA
Number of nodes	48	36	50	39	35	50	49	48
Number of edges	100	100	100	100	100	100	100	100
Modularity	0.325	0.207	0.58	0.473	0.426	0.538	0.593	0.64
Number of communities	3	3	3	3	3	3	3	3
Network diameter	2	2	2	2	2	2	2	2
Network density	0.089	0.159	0.082	0.135	0.168	0.082	0.085	0.089
Average shortest path length	1.822	1.708	1.759	1.71	1.611	1.746	1.746	1.732
Average clustering coefficient	0.862	0.836	0.835	0.876	0.864	0.814	0.85	0.881
Positive correlation (%)	53	53	55	53	51	56	58	60
Negative correlation (%)	47	47	45	47	49	44	42	40

To further investigate microbial interactions and identify potential core microorganisms, a bacterial Zi-Pi analysis was conducted, as outlined in Table 3 and Supplementary Figure S3. Microorganisms classified as connectors are typically keystone taxa that exert significant roles in molding the entire microbial community. For bacteria, the CK, HM, TA, and TC samples contained 4, 1, 3, and 1 connector nodes, respectively. The keystone taxa for HM soil was Acidobacteriota, while Gemmatimonadota was identified as the keystone taxa for TC soil. In TA soil, the keystone taxa included Acidobacteriota, Proteobacteria, and unclassified_Bacteria. At the genus level, Sphingomonas was identified as a keystone taxon exclusive to TA soil among the three STPs. For fungi, the CK, HM, TA, and TC samples contained 2, 2, 2, and 3 connector nodes, respectively. The keystone taxa for HM soil fungi were unclassified_Fungi and Mortierellomycota, while Glomeromycota, Basidiomycota, and Ascomycota were the keystone taxa for TC soil. In TA soil, the keystone taxon was Ascomycota. The results show that different STPs attracted different microorganisms to be connector to assisted the host plant in alleviating salt-induced harm.

**Table 3:** Identification of keystone nodes in bacterial and fungal networks.

		Bacteria		Fungi	
		Phylum	Genus	Phylum	Genus
CK	Module hubs	Proteobacteria	unclassified_ *Geminicoccaceae*	Mortierellomycota	*Mortierella*
		Acidobacteriota	unclassified_ *Vicinamibacteraceae*	Glomeromycota	unclassified_ Glomeraceae
		unclassified_ Bacteria	unclassified_Bacteria		
		Gemmatimonadota	unclassified_ *Gemmatimonadaceae*		
HM	Module hubs	Acidobacteriota	unclassified_ *Vicinamibacterales*	unclassified_Fungi	unclassified_Fungi
				Mortierellomycota	*Mortierella*
TC	Module hubs	Gemmatimonadota	unclassified_ *Gemmatimonadaceae*	Glomeromycota	*Funneliformis*
				Basidiomycota	unclassified_ *Basidiomycota*
				Ascomycota	*Alternaria*
TA	Module hubs	Acidobacteriota	unclassified_ *Vicinamibacterales*	Ascomycota	*Schizothecium*
		Proteobacteria	*Sphingomonas*	Ascomycota	unclassified_ *Stachybotryaceae*
		unclassified_ Bacteria	unclassified_Bacteria		

### 3.4 Functional shifts in soil microbial communities under phytoremediation

**Figure 4:**
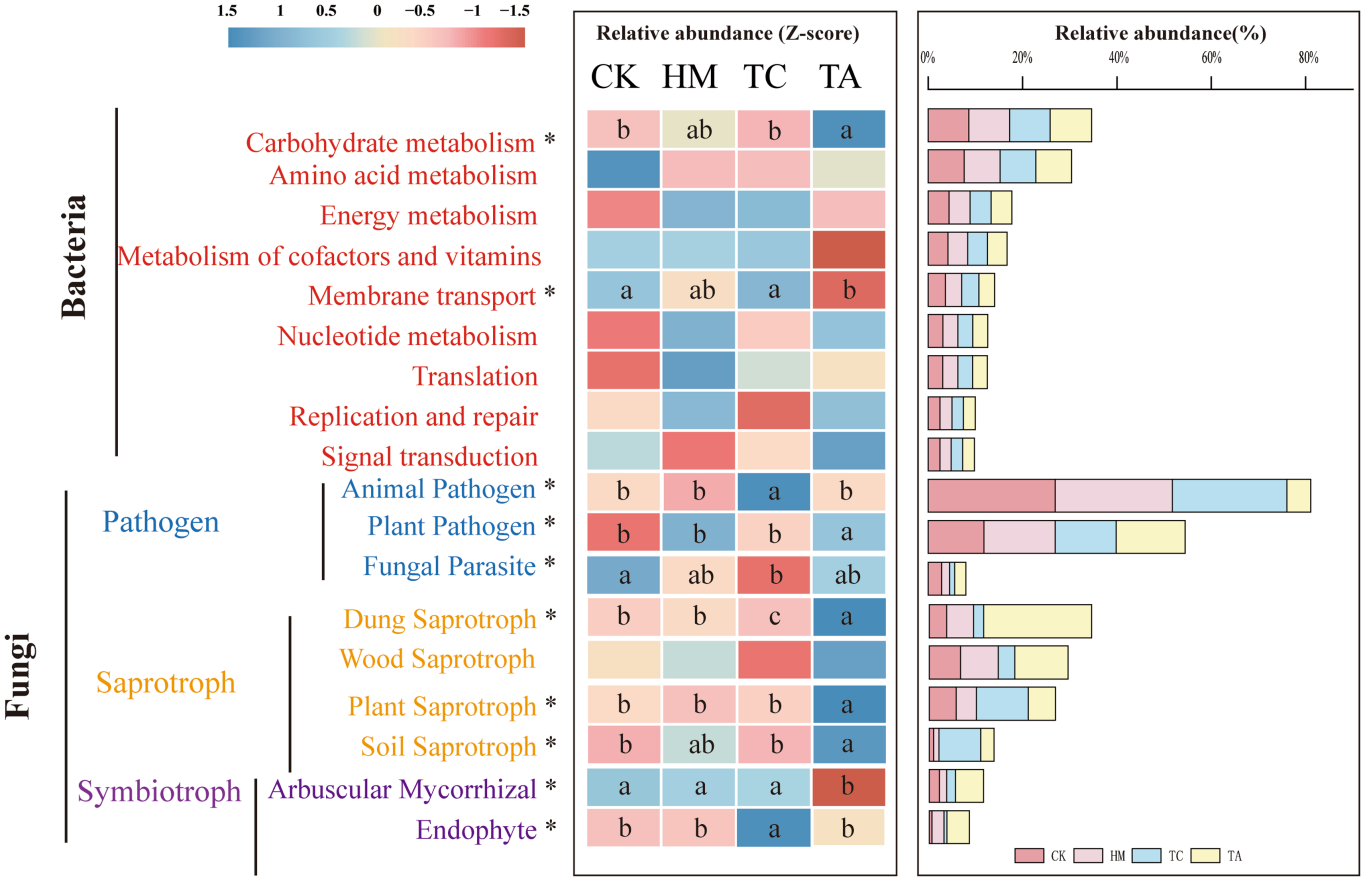
Relative abundance of functional annotations in soil samples of different STPs. Diferent lowercase letters indicate signifcant diferences, ANOVA, Duncan test, *p* < 0.05. The letter sequence (a > ab > b > bc > c) corresponds to descending order of mean values.

The main functional categories at KEGG Level 1 included Metabolism (78.99%), Genetic Information Processing (78.99%), environmental information processing (6.00%). The results indicated that metabolism serves as the primary core function within the bacterial community. A total of 44 functional genes were annotated at Level 2 (Supplementary Figure S4A). Figure 4 displays the functions of the top nine genes. Among the treatments, TA significantly increased carbohydrate metabolism compared to CK, whereas it significantly decreased membrane transport relative to CK (*p* < 0.05).

Soil fungi were categorized into three nutrient modes: pathotroph, symbiotroph, and saprotroph. Among the tested samples, saprotrophs were the predominant nutrient type (49.03%) (Supplementary Figure S5A). A total of 25 functions were identified through functional prediction of fungi (Supplementary Figure S5B), with the top nine features displayed in Figure 4. Compared to CK, the abundance of functional genes associated with Dung Saprotrophs and Fungal Parasites in TC soil exhibited a marked decline (*p* < 0.05), while the abundance of functional genes related to Animal Pathogens and Endophytes significantly increased (*p* < 0.05). The abundance of functional genes associated with Plant Pathogens, Dung Saprotrophs, Plant Saprotrophs, and Soil Saprotrophs in TA soil displayed a notable increase (*p* < 0.05). Conversely, the abundance of functional genes linked to Arbuscular Mycorrhizae exhibited a notable decline (*p* < 0.05). No substantial changes in gene function were noticed between HM and CK. These findings indicate that variations in soil microbial functions are evident due to differences in plant species. Specifically, TC increased the abundance of endophytic fungi while decreasing the abundance of saprophytic fungi, while TA enhanced the abundance of saprophytic fungi but reduced the relative abundance of symbiotic fungi.

### 3.5 Correlation between microbial community changes and soil quality improvements

**Figure 5:**
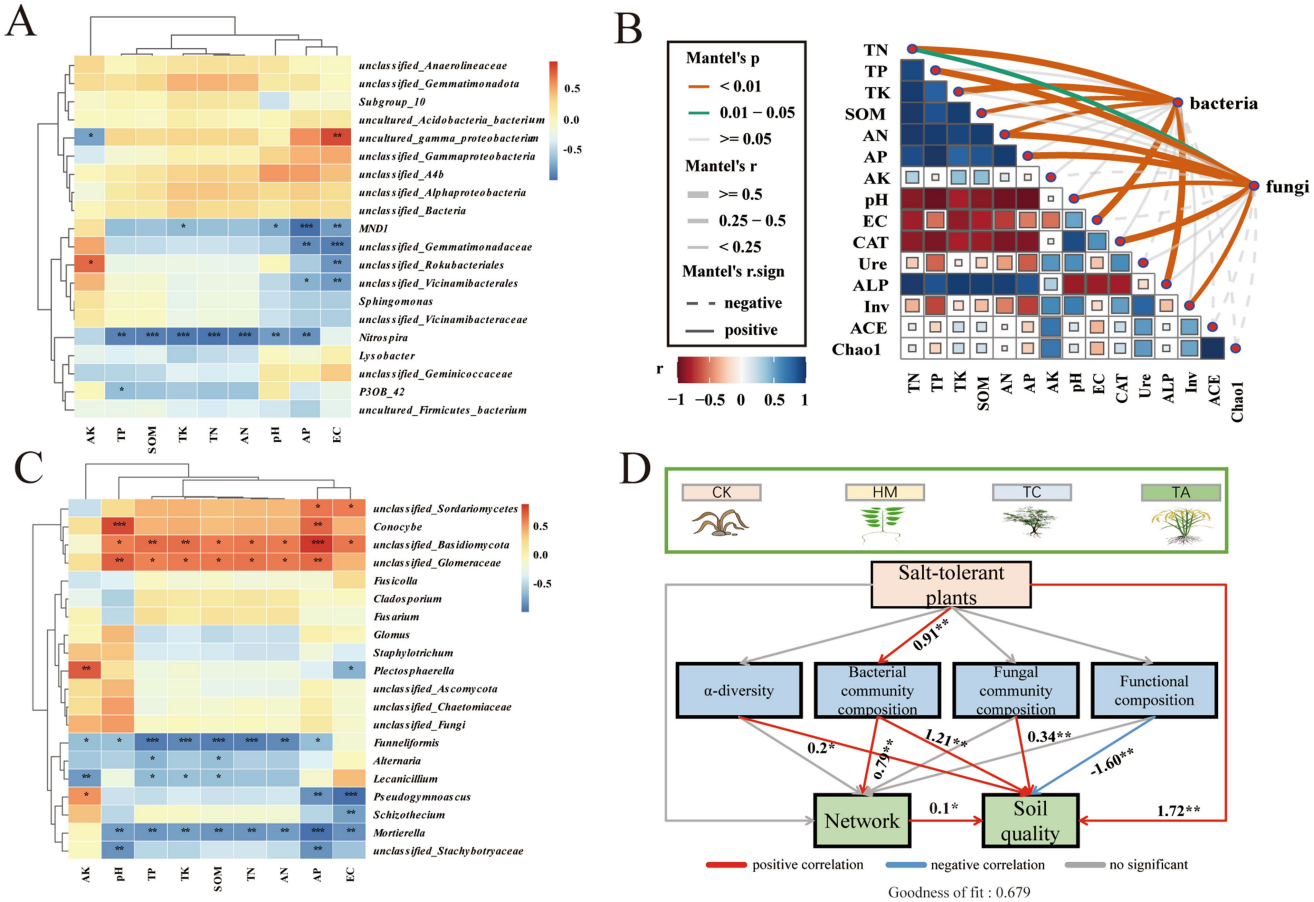
Correlation between soil properties and microbial diversity: **(A)** heatmap of correlations between environmental factors and bacterial genera, **(B)** Mantel test of environmental variables and microbial diversity, **(C)** heatmap of correlations between environmental factors and fungal genera, **(D)** A partial least squares path model (PLS-PM) of microbial composition, functional changes, and soil quality.

To evaluate the impact of environmental factors upon microbial communities, the Mantel analysis and heat map analysis were conducted between environmental factors and microbial flora (Figure 5). Mantel analysis revealed that the abundance of bacteria was highly correlated with TN, TP, TK, SOM, AN, AP, EC, ALP (*p* < 0.01). The fungi abundance had a significant correlation with TN, TP, AN, AP, pH, CAT, and Inv (*p* < 0.05; Figure 5B). Heat map analysis showed that the keystone taxa unclassified_*Gemmatimonadaceae* and unclassified_*Vicinamibacterales* of bacteria were significantly negatively correlated with EC and AP (Figure 5A). *Alternaria* was negatively correlated with SOM and TP, *Schizotheciumi* was negatively correlated with EC. The unclassified_*Basidiomycota* were positively correlated with most of the environmental factors (Figure 5C). These findings indicate that keystone taxa are significantly influenced by soil environmental factors in saline-alkali soils. PLS-PM analysis revealed that STPs had direct effects on soil quality and indirect effects on the microbial network (Figure 5D). Specifically, STPs enhance soil quality by inducing changes in microbial diversity, taxonomic associations, and functional groups of soil biota. Despite the negative impact of microbial functional groups on soil quality, the planting of STPs exerted a positive influence on soil quality and the microbial network primarily through alterations in the bacterial community composition.

## 4 Discussion

### 4.1 Soil quality improvement through STP-driven modifications in physical and chemical properties

In this study, three STPs (TC, TA, and HM) effectively reduced soil EC and pH while increasing soil nutrient levels and enzyme activity. This is consistent with the results obtained by Hu et al. using *Bassia scoparia* and Suaeda salsa to reduce soil pH and salinity (Hu et al., 2024). These findings highlight the potential of TC, TA, and HM as viable options for improving saline-alkali soil. Recent studies suggested that the reduction in EC through the cultivation of STPs may result from the absorption and accumulation of saline ions (Huang et al., 2024). For instance, wild soybeans can absorb approximately 264.57 kg of soluble salinity from saline soil per hectare annually (Xu et al., 2020). Additionally, the excretion of acidic substances by STPs via their root systems can lead to a decrease in pH, facilitating a transition from alkaline to neutral or even more acidic conditions. This pH adjustment enhances nutrient uptake and utilization for plants (Cao et al., 2024).

However, the improvement of the physicochemical properties of saline-alkali soil differs in the three types of STPs. This phenomenon arises from the differing adaptive mechanisms of various plant species to salt-induced stress. Among the three STPs, TA demonstrated the most significant improvement in the physicochemical characteristics of saline-alkali soil. The salt tolerance mechanism of TA primarily involves maintaining a high K^+^/Na^+^ ratio and Ca^2+^/Na^+^ ratio to mitigate ion stress, reducing levels of reactive oxygen species, and accumulating osmoregulatory substances such as proline and soluble proteins to withstand osmotic stress (Hayat et al., 2020). Research has identified numerous genes associated with salt stress in TA, including TaARG, TaSC, TaHKT, and TaPTF1, which significantly enhance its salt tolerance (Aycan et al., 2021). In addition, TA exhibited the highest alkaline phosphatase and invertase activities among the three salt-tolerant plants, indicating its significant potential in enhancing soil fertility. Therefore, TA emerges as a promising STP for improving saline-alkali soil.

### 4.2 Alterations in microbial community composition and function contributing to soil remediation

The strong positive correlation identified between microorganism communities and environmental factors in this study implied that planting STPs could exert a favorable influence on microorganisms and consequently contribute to the amelioration of saline-alkali soil. It was observed significant differences in bacterial enrichment in saline-alkali soil among the three STPs, leading to alterations in the functionality of soil bacteria. Both HM and TA notably decreased the abundance of Proteobacteria while increasing the abundance of Acidobacteriota. The Proteobacteria phylum is predominant in many soil environments, particularly in saline-alkali soils, indicating a relatively abundant presence of salt-tolerant microbial flora within this phylum (Zheng et al., 2017). Furthermore, this phylum includes various nitrogen-fixing bacteria, which play a vital part in the nitrogen cycling process in the soil (Kim et al., 2021). Despite the reduction in HM and TA, Proteobacteria remained the dominant group. Conversely, Acidobacteria, which primarily consist of acidophilic bacteria, are capable of decomposing plant residues and enhancing soil carbon cycling (Jiang et al., 2024). The abundance of Acidobacteria exhibited an inverse correlation with pH (Yao et al., 2024). Wang et al. (2020) used *Lycium chinense* Mill. (LCM), *Tamarix chinensis* Lour. (TCL), and *Gossypium hirsutum* Linn. (GHL) to reclaim saline-alkali soil, which also significantly increased the relative abundance of the phylum Acidobacteria. Through phytoremediation, the increase in Acidobacteria abundance suggests that phytoremediation effectively reduces soil pH and improves saline-alkali conditions. Additionally, the relative abundance of carbohydrate metabolism was significantly higher in TA soils compared to CK soils. Carbohydrate metabolism is related with nitrogen fixation and phosphorus dissolution, promoting the uptake of nitrogen and phosphorus by plant roots (Wang et al., 2024). In addition, carbohydrate metabolism can generate organic acids such as citric acid through TCA cycle, directly reduce soil pH, and chelate Na^+^, while providing carbon sources for microorganisms, activating their Na^+^/H^+^ reverse transport genes, and reducing intracellular Na^+^ accumulation (Zhou et al., 2023). This results show STPs can promote soil nutrient cycle and reduce the harmful impact of salt ions to plant.

Fungal community structure showed, that the abundance of dominant fungal taxa in HM, TC, and TA varied significantly. Different root exudates can result in changes in fungal community structure when different STPs are cultivated. Furthermore, the impact of STPs on fungal community structure was greater than that on bacterial communities. Soil fungi obtain nutrients through a single pathway, whereas bacteria can acquire nutrients via multiple pathways, including autotrophic and heterotrophic methods. The dominant phylum, Mortierellomycota, was significantly enriched in TA soil. Mortierellomycota has the potential to solubilize soil phosphorus by releasing various organic acids (Qu et al., 2022). Given that TA requires more phosphorus, which is vital for nucleic acid and nuclear protein composition, as well as root and fruit formation (Geng et al., 2024; Li et al., 2024), the enrichment of Mortierellomycota in TA soil indicates a positive effect on TA growth. In addition, the relative abundance of saprophytic fungi in TA was significantly increased. This accelerates the decomposition of organic matter, releases nutrient content, and alleviates salt stress. These may contribute to TA’s superior effectiveness among the three STPs in improving saline-alkali soil. Combined with structural equation modeling analysis, STPs enhanced soil quality chiefly through modifying the makeup and function of the soil microbial community.

### 4.3 Enhanced microbial network complexity as a key mechanism in phytoremediation

Compared to CK, the number of nodes, average path length, and modularity of fungi increased in STPs, indicating that planting STPs enhances the complexity of fungal networks. Liu Z. et al. (2023) also found that the rhizosphere of STPs exhibited more complex fungal co-occurrence networks, which aligns with our results. This was further supported by the presence of generalists (connectors and module hubs) in the saline-alkali soils of phytoremediation (Supplementary Figure S3). Generalists can connect with multiple microbial species both within their module and across other modules (Sun et al., 2024). In addition, the composition of generalists in bacterial and fungal networks differed, with a higher proportion of generalists observed in fungal networks (Table 3). Phytoremediation improves the stability and efficiency of fungal communities.

Microbial keystone taxa have a significant impact on soil enzyme activity and nutrient cycling (Wen et al., 2024). For example, certain members of the Proteobacteria can efficiently secrete urease, catalase, and invertase, which promote nitrogen cycling, antioxidant capacity, and carbon source supply, respectively. In contrast, members of the Acidobacteriota and Ascomycota can enhance phosphorus availability by secreting phosphatase. Compared to CK, the number of keystone bacterial taxa in the three STPs decreased, indicating that STPs can alter the microbial network. Additionally, differences in keystone taxa among the three STPs further emphasize the varied effects of each STP on the microbial network. *Sphingomonas* was identified as a keystone taxon exclusive to TA soil. *Sphingomonas* has been documented to promote plant growth and enhance resistance to abiotic stresses, including salinity (Luo et al., 2023). This could also contribute to TA’s more effective improvement of saline-alkali soil compared to HM and TC.

This research also revealed that both the repaired and unrepaired soils showed a greater proportion of positive correlations compared to negative ones within the microbial networks (Figure 3). Generally, the presence of positive interactions between different taxa within a network signifies cooperative behavior (Xue et al., 2020). In nutrient-scarce conditions, microorganisms are inclined to collaborate, potentially enhancing their collective stability (Liu C. et al., 2023). Thus, when faced with nutritional limitations, soil microbes tend to cooperate more than compete, often establishing symbiotic relationships to obtain essential nutrients and mitigate salt stress (Wen et al., 2024). Notably, the ratio of positive correlations within the fungal networks of the three soil types post-phytoremediation exceeded that observed in CK. These findings indicate that phytoremediation strengthens cooperative interactions among fungi and diminishes competitive dynamics.

This study found that STPs can significantly improve the physical and chemical properties of saline-alkali soils and the structure of microbial communities, thereby enhancing soil fertility. These findings provide important theoretical basis and practical guidance for the ecological restoration of saline-alkali lands and the development of sustainable agriculture. For instance, TA significantly promoted the relative abundance of specific microbes (such as *Sphingomonas*) in the soil, which can decompose organic matter, fix nitrogen, and solubilize phosphorus, thus improving soil fertility. Therefore, the combined application of salt-tolerant plants with these beneficial microbes can serve as an efficient soil improvement strategy. To apply these findings to actual agricultural ecosystems, we recommend conducting larger-scale field trials to verify the effects of the synergistic action of salt-tolerant plants and microbial communities under different soil types and environmental conditions. In addition, the inherent differences among plants themselves (such as growth rate, root structure, and metabolic products) may also exert an impact on microbial communities. Future research could further explore these effects by including more plant species, controlling plant growth stages, and conducting long-term monitoring.

## 5 Conclusion

The cultivation of salt-tolerant plants (TC, TA, and HM) effectively reduced the EC and pH of saline-alkali soils while enhancing nutrient content and enzyme activity. Among the three STPs, TA demonstrated the most substantial improvement in soil quality, potentially due to the enrichment of *Sphingomonas* within the microbial community. This study innovatively used network analysis to explore microbial community dynamics. The findings reveal that STP planting not only restructures soil microbial diversity but also promotes beneficial microbial interactions and symbiotic relationships, fostering a more complex and resilient microbial ecosystem. PLS-PM further clarified that the improvement in soil quality was primarily driven by changes in bacterial community composition, underscoring the importance of microbial communities in soil remediation. These results highlight the potential of STPs to mitigate the negative impacts of soil salinity on microbial communities, providing new strategies for managing saline-alkali soils sustainably. However, this research is limited by its short-term nature. Future studies should focus on long-term field trials to evaluate the long-term effects and explore the specific metabolic pathways in plant-microbe interactions for a better understanding of soil improvement and plant tolerance mechanisms.

## Data Availability

The original contributions presented in the study are publicly available. Bacterial original sequence submitted to NCBI with registration number PRJNA1224558. Fungal original sequence submitted to NCBI with registration number PRJNA1228179.
