# Supplementary material for: Microbial network-driven remediation of saline-alkali soils by salt-tolerant plants
Source: Front Microbiol. 2025 Apr 9;16:1565399. doi: 10.3389/fmicb.2025.1565399 (PMC12017681; doi:10.3389/fmicb.2025.1565399)
Supplement: Supplementary file 1 [file Data_Sheet_1.docx]

***Supplementary Data***

Microbial Network-Driven Remediation of Saline-Alkali Soils by Salt-Tolerant Plants

Yushuang Cui^1,†^, Zhifang Ning^1,†^, Menglu Li^1^, Xue Qin^1^, Xin Yue^1^, Xiaobo Chen^2^, Changxiong Zhu^1^, Hongyong Sun^3^, Yali Huang^1,*^

^1^ College of Environmental Science and Engineering, Hebei University of Science and Technology, Shijiazhuang, 050018, China

^2^ College of Food Science and Biology, Hebei University of Science and Technology, Shijiazhuang, 050018, China

^3^ Center for Agricultural Resources Research, Institute of Genetics and Developmental Biology, Chinese Academy of Sciences

**^†^** These authors contributed equally to this work and should be considered co-first authors

*** Correspondence:**

Corresponding author: College of Environmental Science and Engineering, Hebei University of Science and Technology, Shijiazhuang, 050018, China

E-mail address: huangyali2291@163.com (YH)


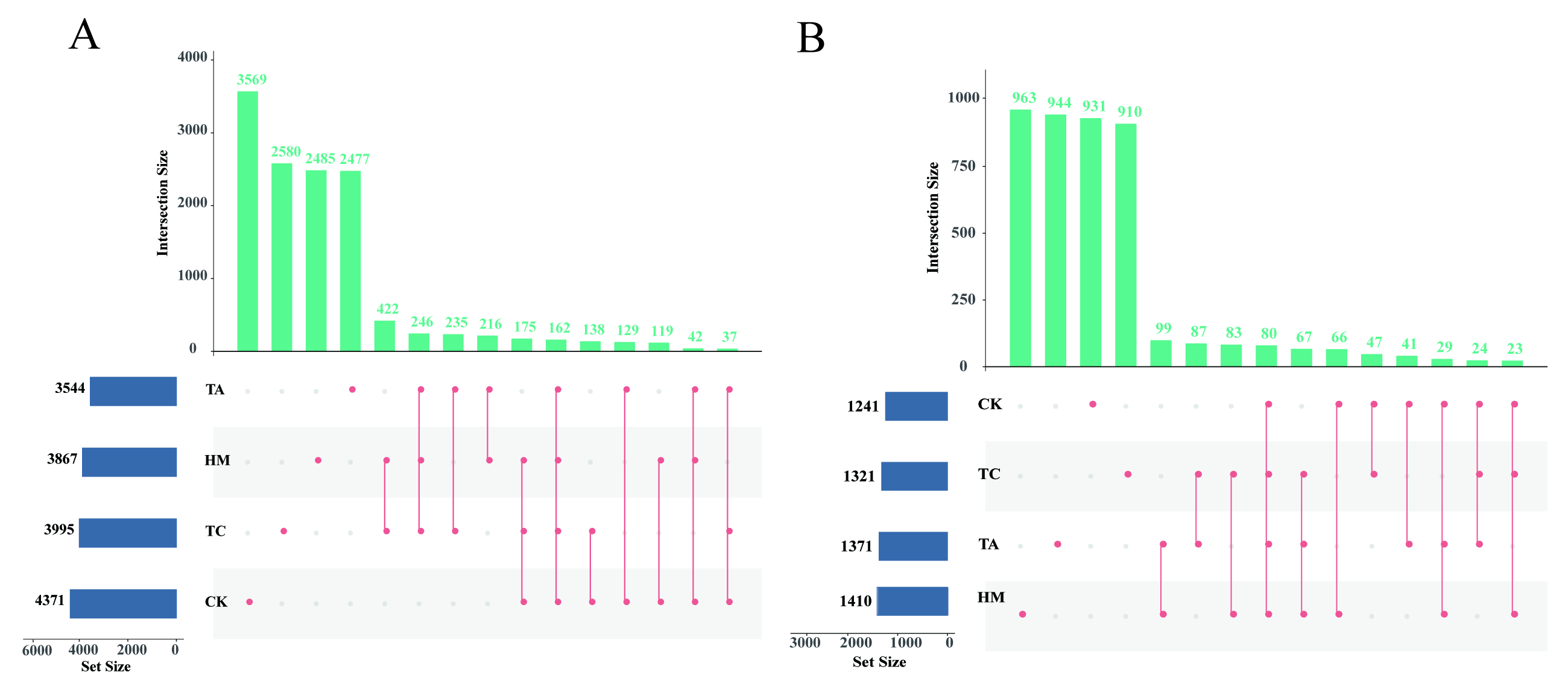


**Figure S1** UpSet Venn diagram representing the number of ASV detected in all samples from different treatments. A) bacterial and B) fungal.


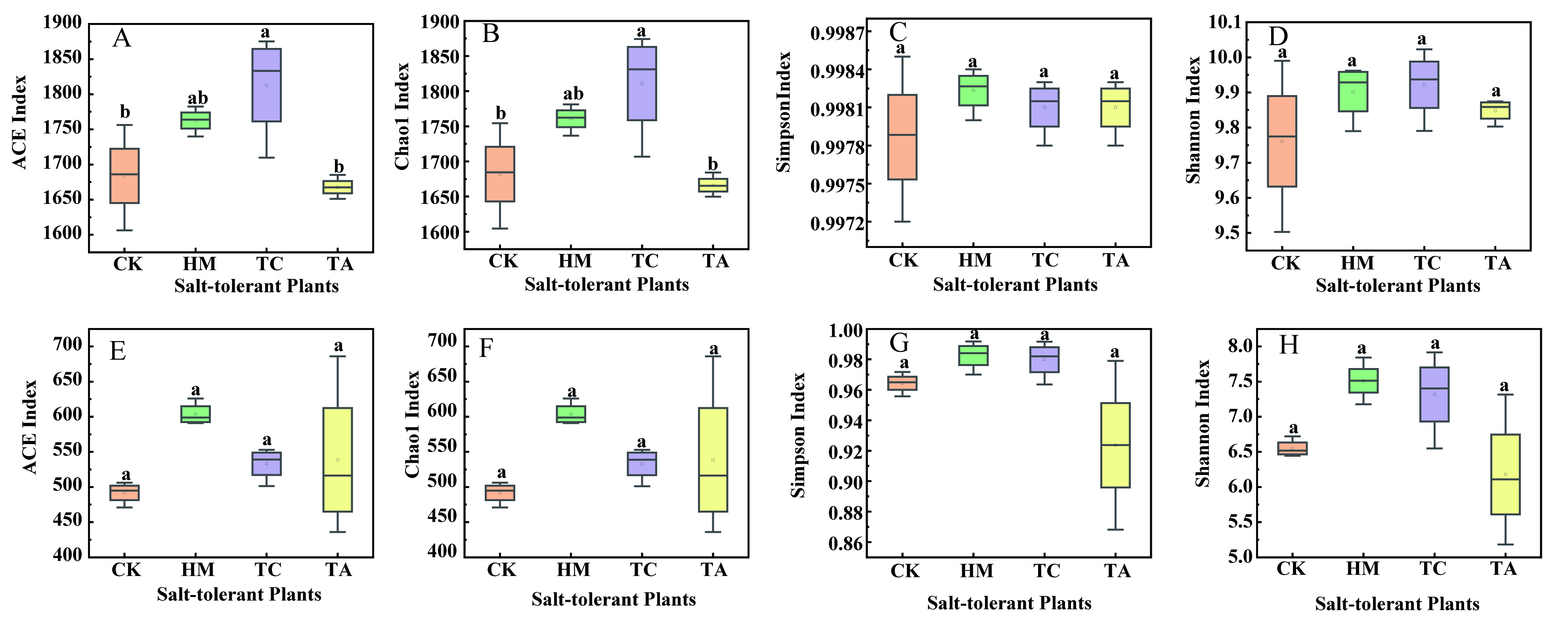


**Figure S2** Effects of different STPs on soil α diversity. A) ACE, B) Chao 1, C) Simpson, D) Shannon indices of bacteria; E) ACE, F) Chao 1, G) Simpson, H) Shannon indexes of fungi. Diferent lowercase letters indicate signifcant diferences, ANOVA, Duncan test, p<0.05. The letter sequence (a > ab > b > bc > c) corresponds to descending order of mean values.


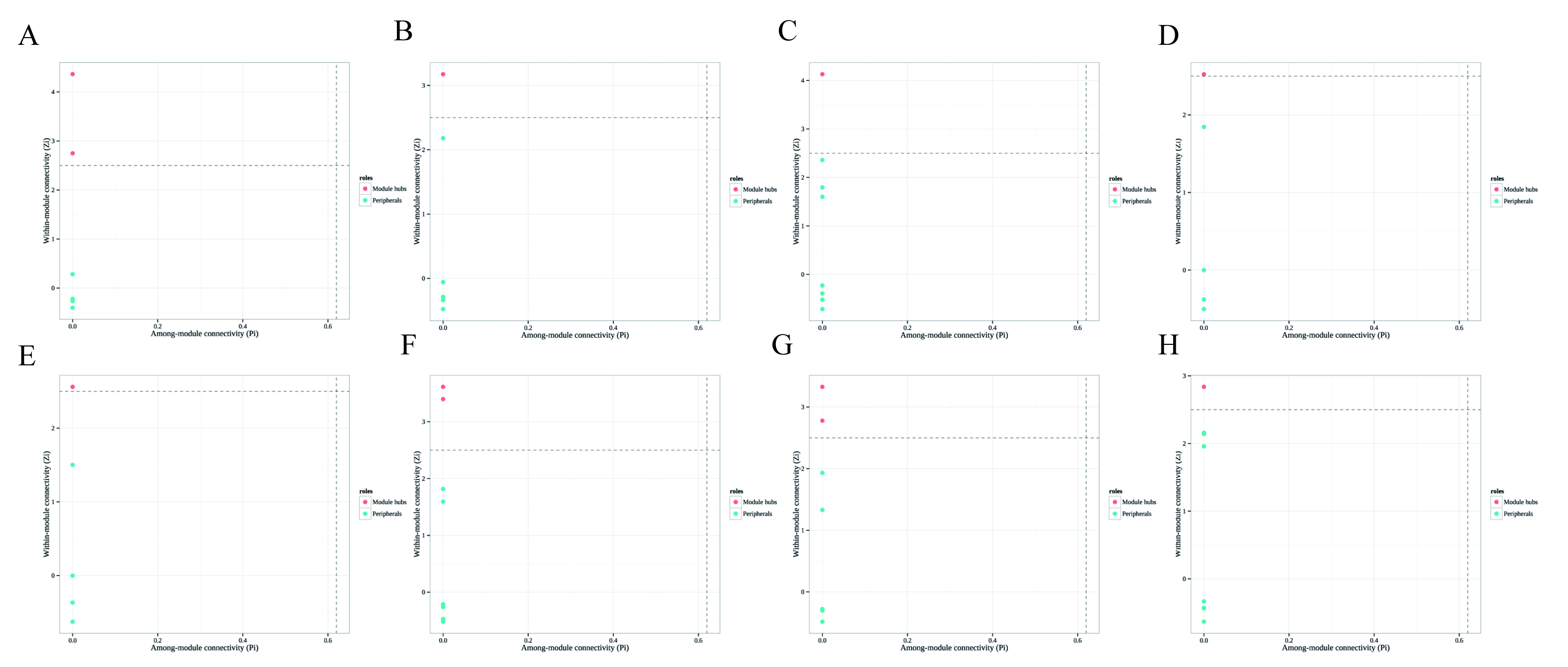


**Figure S3** Zi-Pi analysis of bacteria in A) CK, B) HM, C) TC, D) TA, and fungi in E) CK, F) HM, G) TC, H) TA.


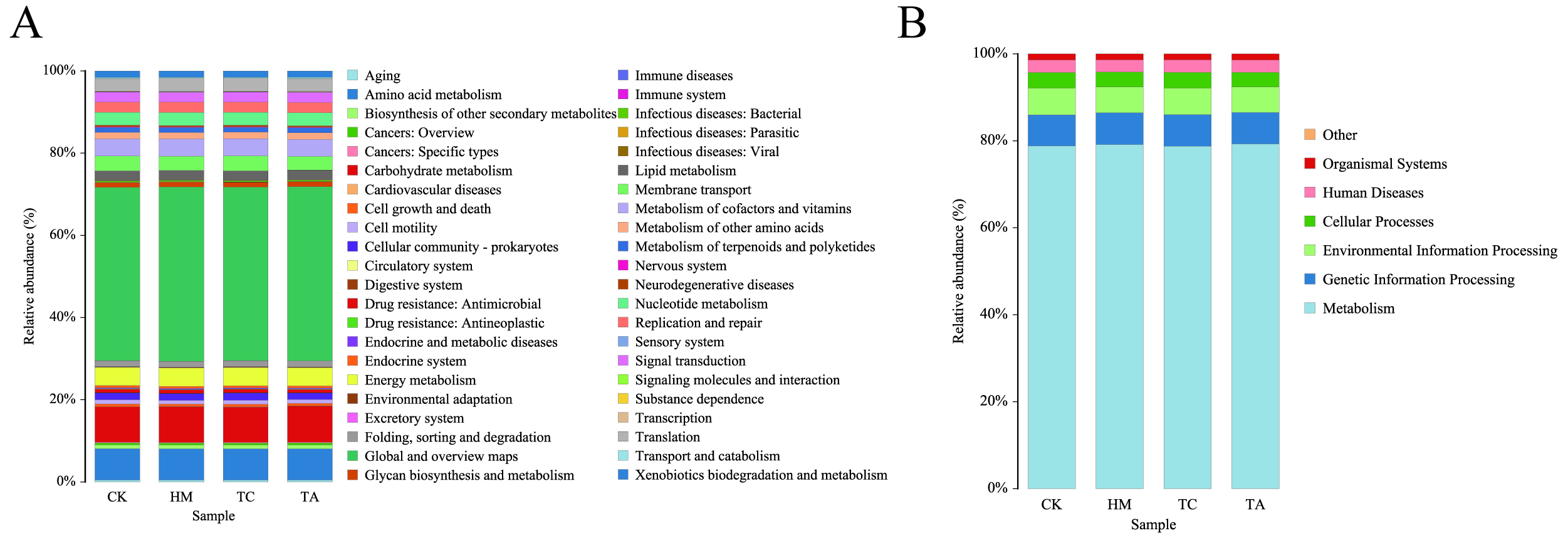


**Figure S4** Relative abundance of functional annotations in soil samples of different STPs. A) and B) bacterial PICRUSt function annotation.


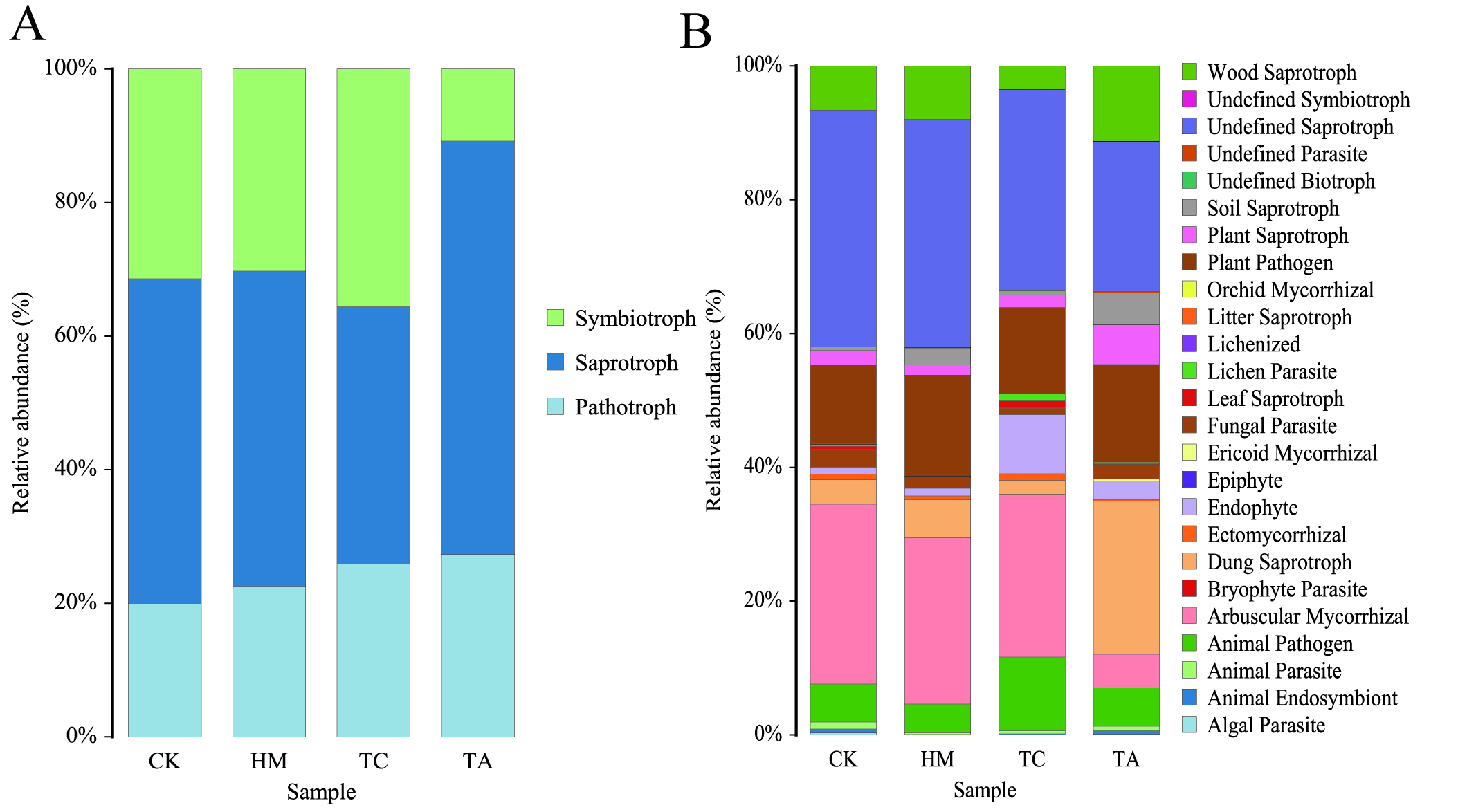


**Figure S5** Relative abundance of functional annotations in soil samples of different STPs. A) and B) FunGuild function annotation.
